# Case report of novel *DYRK1A* mutations in 2 individuals with syndromic intellectual disability and a review of the literature

**DOI:** 10.1186/s12881-016-0276-4

**Published:** 2016-02-27

**Authors:** Stephanie M. Luco, Daniela Pohl, Erick Sell, Justin D. Wagner, David A. Dyment, Hussein Daoud

**Affiliations:** Department of Genetics, Children’s Hospital of Eastern Ontario, Ottawa, ON K1H 8L1 Canada; Division of Pediatric Neurology, Children’s Hospital of Eastern Ontario, Ottawa, K1H 8L1 ON Canada; Children’s Hospital of Eastern Ontario Research Institute, Ottawa, K1H 8L1 ON Canada

**Keywords:** *DYRK1A*, Intellectual disability, Microcephaly, Next Generation Sequencing

## Abstract

**Background:**

Chromosomal deletions encompassing *DYRK1A* have been associated with intellectual disability for several years. More recently, point mutations in *DYRK1A* have been shown to be responsible for a recognizable syndrome characterized by microcephaly, developmental delay and intellectual disability (ID) as well as characteristic facial features. Here we present 2 individuals with novel mutations in *DYRK1A*, and a review of the cases reported to date.

**Case presentation:**

Both individuals presented with the well-known characteristic features, as well as rarer anomalies seen in a minority of patients. Patient 1 presented shortly after birth with an enlarged cisterna magna, distal contractures, and distinctive facies that included bitemporal narrowing and deep set eyes. A *de novo* splice site mutation in *DYRK1A* [c.951 + 4_951 + 7delAGTA; p.Val222Aspfs*22] was identified by next generation sequencing. Patient 2 presented at 7 months of age with microcephaly and dysmorphic features. She went several years without a diagnosis until a *de novo DYRK1A* nonsense mutation [c.787C>T; p.(Arg263*)] was identified at age 12. These individuals, and the 52 cases reviewed from the literature, show the characteristic features of the *DYRK1A*-related syndrome including global developmental delay, ID, microcephaly, feeding difficulties, and the facial gestalt. Other common findings include seizures, vision defects, brain abnormalities and skeletal abnormalities of the hands and feet. Less common features include optic nerve defects, contractures, ataxia, and cardiac anomalies.

**Conclusion:**

*DYRK1A* testing should be considered in individuals with the facial features, intellectual disability and post-natal microcephaly. Once diagnosed with *DYRK1A*-related intellectual disability, a cardiac and ophthalmologic assessment would be recommended as would routine surveillance by a pediatrician for psychomotor development, growth, and feeding.

## Background

The dual-specificity tyrosine phosphorylation-regulated kinase 1A (*DYRK1A*) (MIM 600855) is located in the Down syndrome critical region of chromosome 21 [1]. It encodes a highly conserved protein that plays an essential role in the development of the central nervous system [2–4]. Haploinsufficiency of *DYRK1A* is responsible for a syndrome characterized by intellectual disability (ID), microcephaly and dysmorphic features (MIM 614104). Individuals with this condition were initially reported with partial monosomies of chromosome 21 as detected on routine karyotype that encompassed the *DYRK1A* gene (21q22.13) [5–7]. Since then, numerous cases of chromosome 21 deletions diagnosed by comparative genomic hybridization have been reported by several groups [8–13]. The first intragenic frameshift deletion, identified by direct sequencing, was described by Courcet et al in 2012 [14]. More recently, next generation sequencing (NGS) has led to the diagnosis of numerous *de novo* mutations in *DYRK1A*, further characterizing, and broadening, this syndromic phenotype [15–21]. To date, there have been 52 individuals in the literature with the *DYRK1A*-related ID phenotype with both structural rearrangements (*n* = 19) and more recently, single nucleotide variants and small insertions/deletions within *DYRK1A* (*n* = 33).

Here, we present 2 additional patients with novel *DYRK1A* mutations. We also provide a comprehensive review of the previously published cases, further delineating both the common and less commonly seen features of this emerging syndrome.

## Case presentations

**Fig. 1:**
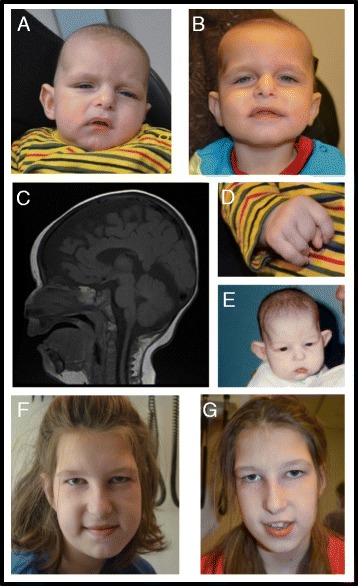
The characteristic facies of 2 individuals with *DYRK1A* mutation. **a**-**b** Patient 1 with bitemporal narrowing, down-slanted palpebral fissures, deep-set eyes and dysplastic ears. **c** MR Images in Patient 1 showing thin corpus callosum, enlarged cisterna magna and 4th ventricle. **d** distal contractures seen in Patient 1. **e**-**g** Patient 2 with with bitemporal narrowing, deep-set eyes and dysplastic ears

Patient 1 was born at 36 + 3 weeks, to healthy parents of Iraqi origin. Parents were first cousins once removed. Two older siblings were in good health. There was no family history of ID or congenital anomalies. Intrauterine growth restriction (IUGR) and an enlarged cisterna magna were observed at 35 weeks by routine ultrasound. A prenatal diagnosis of Dandy-Walker malformation was considered but ruled out with post-natal MR imaging (Fig. 1c). Labor was induced given poor fetal growth and the patient was born by vaginal delivery at 36 + 3 weeks. At birth, APGAR scores were 6 and 8 at 1 and 5 min. Birthweight was 2.33 kg (−2.5 SD) and head circumference was 31.5 cm (−2.5 SD). On examination he had a sacral dimple, increased appendicular tone, and bilateral contractures to the third and fifth digits (Fig. 1d). An echocardiogram showed a patent ductus arteriosus. He experienced feeding difficulties in the neonatal period and required nasogastric feeding.

MRI at 2 weeks of life showed mild prominence of lateral ventricles and extra-axial spaces, enlarged cisterna magna, and a thin corpus callosum. MRI repeated at 10 months of age showed interval enlargement of the supratentorial extra-axial spaces, mild dilatation of the lateral and 3rd ventricles as well as the enlarged cisterna magna and thin corpus callosum (Fig. 1c).

The patient’s facial features showed bitemporal narrowing with down-slanted palpebral fissures, deep-set eyes with hooded appearance, a prominent nasal root, low-set and mildly dysplastic ears, and a downturned mouth (Fig. 1a, b). He had bilateral ankle contractures and proximal placement of the first toes. At 4 months of age he continued to have contractures of ankles and 3rd digits of his hands. He is currently followed for visual impairment with bilateral optic atrophy. He has bilateral hydronephrosis. He further has a history of recurring otitis media with resultant conductive hearing loss. From a young age, he has experienced frequent dysphagia and emesis with ingestion of thicker liquids. At 15 months, his weight was 7.9 kg (−3.3 SD) and length was 72.9 cm (−2.2 SD). Head circumference showed relatively slower growth velocity at 41.5 cm (−4.6 SD). At 21 months of age, he had yet to stand or say his first words. A karyotype and microarray were normal. Given the history of consanguinity and the syndromic features in Patient 1, the parents of the patient had genetic counselling and were quoted a recurrence risk of up to 25 %. The patient was enrolled in a research study at the Children’s Hospital of Eastern Ontario, at the age of 13 months.

Patient 2 was born at 41 + 3 weeks by spontaneous vaginal delivery, to healthy French Canadian parents. Parents were not consanguineous. The patient has two older siblings in good health. Birthweight was 3.2 kg (- 1.1 SD). There were no concerns in the newborn period with the exception of feeding difficulties. At 7 months of age, she was referred to Genetics for microcephaly and dysmorphic features. At that time she weighed 6.65 Kg (−1.5 SD), with length of 65.2 cm (- 1 SD). Head circumference was 39.8 (−2.9 SD). On examination, she had bitemporal narrowing, a prominent occiput and deep set eyes. In addition, she had a pronounced asymmetry of the ears, with her right ear being lower set, anteverted, and with a simple helix (Fig. 1e-g). She underwent bilateral otoplasty at the age of four.

She experienced several febrile seizures starting at 15 months of age. Her seizures continued in the absence of fever, and she experienced a number of generalized tonic-clonic seizures before beginning anticonvulsant medication at 8 years of age. Her seizures have since been well controlled, with low dose treatment with Levetiracetam. An MRI, at 34 months of age, showed ventricles and subarachnoid space in the upper limits of the normal range but with no other structural abnormality.

She said her first word at 32 months. By 8 years of age she had 10 words, and by 11 years, she occasionally spoke in short sentences. She walked at 25 months. At the age of 12, she was described as having a broad-based clumsy gait, and exhibited a mild tremor and ataxia when reaching for objects. As a toddler, she demonstrated stereotypic behavior including hand flapping, tongue thrusting during feedings, as well as an aversion to being touched and to certain textures. Hand flapping has persisted into adolescence. At 11 years of age, she had a height of 159 cm (+2 SD), a weight of 56 Kg (+1.7 SD), and a head circumference of 51 cm (−1.3 SD). During her last assessment at age 12, her features were further described as a long face, bitemporal narrowing, hooded palpebral fissures, deep set eyes, prominent nasal bridge and bulbous tip to her nose, smooth philtrum, wide spaced teeth, tapering fingers and mild pectus excavatum (Fig. 1g).

During her diagnostic work-up she underwent several investigations that included normal karyotype, chromosomal microarray, FISH for 22q11.2, methylation specific MLPA and sequencing of *UBE3A*, sequencing of *MECP1* and *MECP2*, *TCF4*, and mitochondrial DNA whole genome sequencing all yielded normal results.

## Material and methods

Genomic DNA was extracted from peripheral blood lymphocytes using standard methods. The Trusight One Sequencing panel (Illumina) was used to capture 4813 genes deemed to be “clinically relevant”. Enriched libraries from patient 1 and his parents (trio approach) were pooled and sequenced on the MiSeq (Illumina). The detailed protocol has been previously reported [22]. RT-PCR and Sanger sequencing were performed by standard protocols using primers targeting *DYRK1A* (NM_001396.3) coding exon-5 (Ex5F:5′-TTGAGCTCATGAACAAACATGAC-3′) and coding exon-8 (Ex8R:5′-GAGCAGGTGGAATACCCAGA-3′) on RNA extracted from patient and control lymphoblast cell lines.

## Results and discussion

**Fig. 2:**
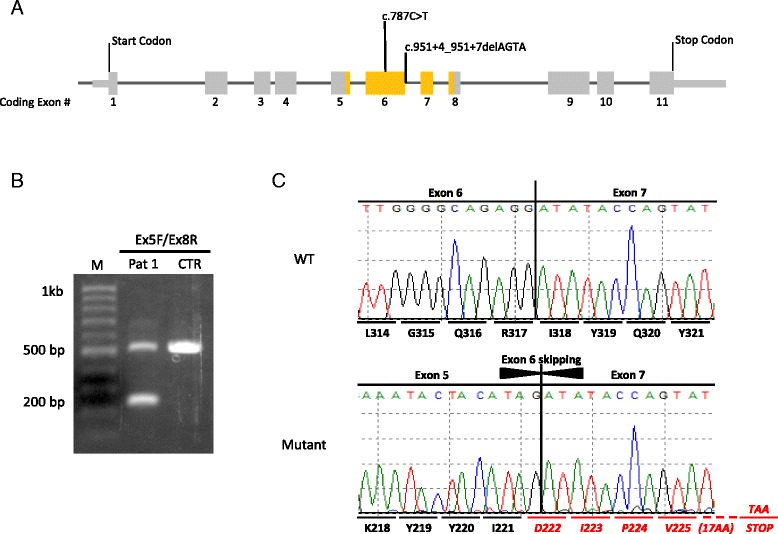
**a** Schematic representation of the coding sequence of *DYRK1A (NM_001396.3)* showing the localization of the two *de novo* mutations identified in this study. **b** Agarose gel electrophoresis of the RT-PCR products from patient 1 (Pat 1) and a control individual (CTR). A fragment covering exons 5–8 was amplified by RT-PCR (*yellow* in **a**). The expected 528 bp PCR product was observed from both patient 1 and the control individual, whereas a smaller PCR product (~240 bp) was only detected in patient 1. A 100 bp ladder was used for reference. **c** Sequencing of both the wild type (WT) and mutant fragments from patient 1 showing the WT *DYRK1A* transcript sequence covering exons 6 and 7, and mutant *DYRK1A* transcript lacking exon 6, resulting in a frameshift and a premature stop codon 22 codons downstream

A *de novo* variant in *DYRK1A* [c.951 + 4_951 + 7delAGTA; p.Val222Aspfs*22] was identified in patient 1 at 14.5 months of age. This variant is predicted to affect splicing by Alamut Visual version 2.7 (Interactive Biosoftware, Rouen, France) and was confirmed by RT-PCR (Fig. 2b) and Sanger sequencing (Fig. 2c). Patient 2 had clinical testing with a next-generation sequencing panel for epilepsy (Comprehensive Epilepsy Panel; Medical Neurogenetics Lab, Georgia, USA) and this revealed a nonsense mutation [c.787C>T; p.(Arg263*)] in *DYRK1A*. Parents were tested and found to not carry the mutation in keeping with a *de novo* mutation.

Both individuals had novel *DYRK1A* mutations, and presented with the key features of the *DYRK1A* phenotype, as well as less commonly observed findings that contribute to a broadening of the phenotypic spectrum of this recognizable syndrome. Patient 1 was seen prenatally given the IUGR and an enlarged cisterna magna observed on routine prenatal ultrasound. At birth, camptodactyly, hypertonia, and distinctive facial features were noted. A diagnosis occurred at 14.5 months of age and was possible because of the early recognition of a likely monogenic syndrome and accessibility to comprehensive, NGS-based panel testing. In contrast, Patient 2 was referred to Genetics given the history of a mild post-natal microcephaly, dysmorphic features and developmental delays. During her childhood she experienced an extensive diagnostic odyssey with many genetic, metabolic, and imaging investigations performed. A molecular diagnosis was also possible with NGS panel testing for epilepsy-related genes at age 12 years.

A PubMed search for reported cases of *DYRK1A* mutations or partial chromosome 21 deletions yielded a total of 52 patients from 17 papers (Table 1). A summary of the clinical features of the 2 patients and the previously published cases is shown in Table 2. We recognize that any conclusions based on published data may enrich for more severe phenotypes; however the relatively large number of cases (*n* = 54) may attenuate this potential source of bias. The age at diagnosis of the patients and those from the literature showed a wide range from 17 months to 59 years. The 14.5 months observed for patient 1 was the earliest reported molecular diagnosis. On history, disease manifestations were early in most patients and prenatal findings were observed in 33/43 (77 %). This included 21 cases of IUGR, 2 cases of oligohydramnios and 1 case of polyhydramnios. Birth measurements were commonly in the low range (<-2 SD), though this was not universal. In aggregate, the most common features were ID and developmental delay seen in 53/53 cases (100 %), dysmorphic facies in 53/54 (98 %), feeding difficulties in 44/46 (96 %), and microcephaly (<-2 SD) seen in 44/51 (86 %). A number of other features were also noted in the majority such as stereotypies and abnormal gait (Table 2). Weight and height at last clinical assessment were less than − 2 SD in only 44 and 42 % respectively. There were 5 patients who were within the normal range for height, weight and head circumference.

**Table 1:** *DYRK1A *mutations reported in the literature

Paper	# of patients	Types of mutations
Bartsch et al 1997 [7]	1	Translocation
Matsumoto et al 1997 [6]	1	Deletion
Møller et al 2008 [8]	2	2 translocations
Fujita et al 2010 [9]	1	Deletion
Oegema et al 2010 [10]	2	2 deletions
Yamamoto et al 2011 [11]	3	3 deletions
van Bon et al 2011 [12]	1	Deletion
Valetto et al 2012 [13]	1	Deletion
Courcet et al 2012 [14]	2	1 deletion, 1 frameshift
O’Roak et al 2012 [15]	3	2 frameshifts, 1 splice site
Okamoto et al 2015 [27]	1	Nonsense
Redin et al 2014 [16]	2	1 nonsense, 1 frameshift
Iglesias et al 2014 [17]	1	Nonsense
Ruaud et al 2015 [19]	2	1 nonsense, 1 missense
van Bon et al 2015 [18]	5	2 nonsense, 3 splice site
Bronicki et al 2015 [20]	10	3 nonsense, 2 missense, 4 frameshift, 1 deletion
Ji et al 2015 [21]	14	3 nonsense, 3 missense, 3 frameshift, 5 deletions

**Table 2:** Summary of the clinical presentation of patients with *DYRK1A* mutations

	Patient 1	Patient 2	SNV, Splice, FS (33)	Rearrangements (19)	Total (54)
Age at last reported assessment	22 months	13 years	21 month - 59 years	17 months - 33 years	
IUGR / prenatal findings	IUGR, enlarged cisterna magna	-	17 / 26	15 / 16	33 / 43
Head circumference at birth (<-2 SD)	- 2.5 SD		13 / 22	15 / 16	29 / 39
Low birth weight (<-2 SD)	- 2.5 SD	-	13 / 27	14 / 19	28 / 47
Length at birth (<-2 SD)	-	-	12 / 21	12 / 15	24 / 36
Feeding difficulties	+	+	28 / 30	14 / 14	44 / 46
Microcephaly (<-2 SD)	- 4.6 SD	- 1.3 SD	26 / 31	17 / 18	44 / 51
Weight (<-2 SD)	- 3.3 SD	+1.7 SD	7 / 26	12 / 17	20 / 45
Height (<-2 SD)	- 2.2 SD	+2 SD	7 / 28	12 / 18	20 / 48
Global developmental delay	+	+	33 / 33	18 / 18	53 / 53
Intellectual disability		+	31 / 32	19 / 19	51 / 52
Speech delay / absence	+	+	32 / 32	17 / 17	51 / 51
Motor delay / late walking	+	+	22 / 22	11 / 12	35 / 36
Abnormal gait		+	18 / 20	7 / 9	26 / 30
Behavioral issues	-	+	29 / 31	9 / 11	39 / 44
Stereotypies	-	+	20 / 25	6 / 9	27 / 36
Autism spectrum disorder		-	14 / 27	2 / 7	16 / 35
Anxiety	-	+	9 / 18	1 / 5	11 / 24
Hyperactivity / ADHD	-	-	5 / 21	3 / 7	8 / 30
Febrile seizures	-	+	17 / 29	13 / 15	31 / 46
Seizures / epilepsy	-	+	14 / 28	13 / 17	28 / 47
Brain abnormalities (MRI)	+	Normal	18 / 24	13 / 16	32 / 42
Enlarged ventricles	+		10	4	22^a^
General / cortical atrophy	+		8	6	17^a^
Thin brainstem	-		2	2	13^a^
Hypoplastic corpus callosum	-		2	4	9^a^
Optic disk/nerve anomaly	Optic disc pallor	-	5	1	7
Vision abnormalities	Visual impairment	-	16 / 21	8 / 9	25 / 32
Dysmorphic facies	+	+	32 / 33	19 / 19	53 / 54
Abnormalities of the hands or feet	Small feet, toe brachydactyly, proximal placement of first toes	Tapering fingers, high arched feet	15 / 16	8 / 10	25 / 28
Abnormalities of the spine or chest	-	Mild pectus excavatum	6 / 8	4 / 7	11 / 17
Contractures	Bilateral: third and fifth digits, ankles	-	3 / -	1 / -	5 / -
Gastrointestinal symptoms	GERD, vomiting	-	13 / -	3 / -	17 / -
Genitourinary	Bilateral hydronephrosis	-	5 / -	3 / -	9 / -
Cardiac	PDA	-	2 / -	5 / -	8 / -
Recurrent infections	+	-	7 / -	3 / -	11 / -

*SNV* single nucleotide variant, *FS* frameshift, *mo* Months, *yrs* Years, *IUGR* intrauterine growth restriction, *SD* standard deviation, *ADHD* attention deficit hyperactivity disorder, *MRI* magnetic resonance imaging, *GERD* gastroesophageal reflux disease, *PDA* patent ductus arteriosus

^a^ One paper (Ji et al [21]) did not assign the type of abnormalities to each patient, but gave a total prevalence for their cohort. These values have been added to our total, but were not included in the group subtotals

Febrile seizures were reported in 31/46 (67 %) cases and a later diagnosis of epilepsy or non-febrile seizures was observed in 60 %. Patient 2 and 8 other patients from the literature were reported to have good control of their seizures with anti-epileptic medication. Two children from the literature were described as having progressively worsening seizures, one presented with seizures shortly after birth and the other was refractory to medication by the age 10 years [11, 13]. Both patients had large deletions encompassing *DYRK1A* in addition to other genes on chromosome 21.

Results of brain imaging were reported for 42 patients from the literature. Thirty-two had abnormal findings (76 %). Enlarged ventricles were described in 22 patients, general or cortical atrophy in 17 patients, a thin brainstem in 13 patients, and a thin corpus callosum in 9 patients. Other less common findings include pituitary stalk hypoplasia in 7 cases, a neuronal migration defect in 1 case, and an enlarged cisterna magna observed in our case (Fig. 1c). An additional less commonly seen feature observed in Patient 1 was bilateral optic disc pallor. Anomalies of the optic disc or optic nerve have been seen in 6 other individuals with *DYRK1A* mutations. Visual abnormalities were seen in 25 of 32 patients, including hyperopia, myopia, strabismus, astigmatism, exotropia, amblyopia, and corneal clouding. There was also retinal detachment in the oldest individual published in the literature, at 59 years.

Dysmorphic features characteristic of a *DYRK1A* mutation were near universal in 53/54 patients. This includes deep set eyes with a hooded appearance to the lateral aspect of the palpebral fissures, a prominent nasal root, bitemporal narrowing, prominent and dysplastic ears. Hand and/or foot abnormalities were also seen in 25 patients that included long tapered fingers, small hands and feet, 5th finger clinodactyly, toe syndactyly, high arched feet, and proximal placement of the first toe. Polydactyly was reported in 2 cases. Spinal or thoracic abnormalities were seen in 11 patients; presenting with pectus excavatum, kyphosis, scoliosis, and abnormal cervical spine. Seventeen patients were reported to have gastrointestinal symptoms, including gastroesophageal reflux disease in 12 patients, 3 of whom required a Nissen fundoplication, recurrent vomiting in 4 patients, pyloric stenosis in 2 patients, and duodenal atresia in 1 patient.

Our summary of the previously reported cases and our 2 cases highlight relatively rare findings within this group. Contractures in the digits or lower limbs were seen in our patient 1 (Fig. 1d) as well as 4 others. An ataxic gait, seen in patient 2, was also described in 5 other individuals. There have been 10 cases in the literature that reported recurring or frequent infections. Cardiac anomalies, including ventricular septal defect (VSD), patent ductus arteriosis (PDA), and aortic valve stenosis or insufficiency, were found in 8 cases. There were 9 reports of genitourinary findings: cryptorchidism in 3 patients, unilateral renal agenesis, pelvic kidney, renal cyst, hypospadia, micropenis, and our patient 1 with bilateral hydronephrosis. Though these cardiac and renal findings are not seen in the majority of patients with *DYRK1A* mutations, they should be considered during monitoring and long-term care.

**Fig. 3:**
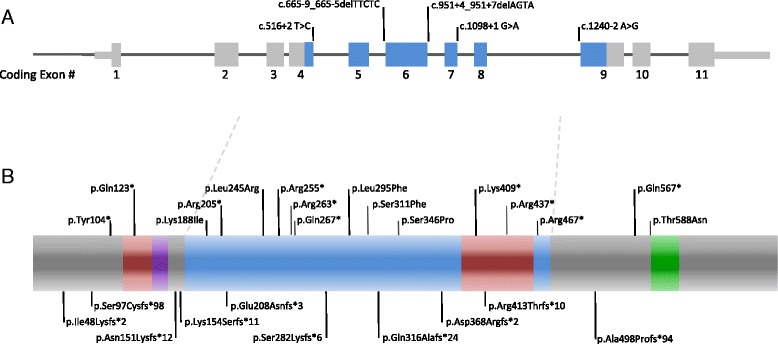
**a** The *DYRK1A* coding sequence with the 5 reported splice site mutations. **b** The DYRK1A protein with the missense and nonsense mutations shown above, and the frameshift variants indicated below. The nuclear localization signal shown in red, the DYRK homology (DH) box shown in purple, the kinase domain shown in blue, and the speckle-targeting signal shown in green. Two mutations were identified in multiple individuals: p.Arg205* in 4 individuals and p.Glu208Asnfs*3 in 2 individuals

A schematic representation of the 35 intragenic and *de novo DYRK1A* mutations is shown in Fig. 3. The DYRK1A protein contains a kinase domain, spanning from residue 158 – 479 [23]. Other important features in the N-terminus include a nuclear localization signal and a DYRK-homology box, a sequence highly conserved in the DYRK family of unknown function [23, 24]. From residue 588 to 616, there is a targeting signal that has been shown to cause localization of the protein to subnuclear compartments associated with splicing machinery [25]. The majority of mutations occurred within the kinase domain or in the N-terminal (12 nonsense, 10 frameshift, and 5 splice site mutations). Of the 6 missense mutations, 5 occurred within the kinase domain, potentially leading to diminished protein function. The only missense mutation falling outside of the functional domain alters the first residue of the speckle-targeting signal, important for the localization of the DYRK1A protein. There does not seem to be a clear genotype-phenotype correlation with the mutations and the phenotypes reported. There were 4 patients with the same nonsense mutation, p.Arg205* reported by different groups and they did not appear to differ from the majority of cases reported. The recurrence of the p.Arg205* mutation is likely due to its location in a CpG dinucleotide, which are mutation hotspots [26].

## Conclusion

In summary, children with ID and syndromic features should be considered for a possible *DYRK1A* mutation testing especially if the recognizable facial features are present. Growth and feeding issues would also support its potential diagnosis. At the time of diagnosis, abdominal ultrasound, cardiac and ophthalmological assessments would be recommended given the frequency of anomalies. Genetic counselling would also be recommended in view of the potential for a balanced translocation in a parent with a child carrying microdeletions encompassing *DYRK1A* [7, 21]. A recurrence due to possible gonadal mosaicism has yet to be observed. Routine follow-up with a pediatrician would be highly recommended given feeding issues, growth and developmental delays.

## Consent

Written and informed consent was obtained from the parents of the patients for the publication of this manuscript and the accompanying images of the patients. A copy of the patient consents for publication of images are available to the Editor of this journal.

The individuals in the study were recruited by physician referral. The study was conducted in accordance with the Declaration of Helsinki protocols and approved by the regional ethics review board at the Children’s Hospital of Eastern Ontario, Ottawa, ON (Protocol Number 08/71X). Written and informed consent for the molecular studies were obtained and available for review by the Editor of the journal.

## Abbreviations

DYRK1A: dual-specificity tyrosine phosphorylation-regulated kinase 1A
ID: intellectual disability
IUGR: intrauterine growth restriction
SD: standard deviation
MR imaging: MRI, Magnetic resonance imaging
DNA: deoxyribonucleic acid
RT-PCR: reverse transcriptase polymerase chain reaction
FISH: fluourescence in situ hybridization
MLPA: multiplex ligation-dependent probe amplification
NGS: next generation sequencing
GERD: gastroesophageal reflux disease
VSD: ventricular septal defect
PDA: patent ductus arteriosus
